# Memoir on Medical Reform

**Published:** 1813-10

**Authors:** 


THE
### Medical and Physical Journal.

4 OF VOL. XXX.] OCTOBER, 1813. [NO. 176.

### *To the Editors of the Medical and Physical Journal.*

GENTLEMEN,

TWO serious attempts have now been made within a short period, to regulate the profession of physic by means of legislative enactments; and it is matter of notoriety, that the associated body whose bill for this purpose has been so lately withdrawn from the table of the House of Commons, have it in contemplation again to introduce the same in an amended form, as soon as parliament shall be re-assembled. The activity hitherto manifested by this body in the pursuit of their favorite scheme, and their well-known habits of industry and application, leave us little room to doubt of their object being pursued with ardour and perseverance.

Under such circumstances, it is surely full time to inquire whether this subject is so thoroughly investigated or understood, as to be prepared for such final appeal; and also, how far they who have come forward to point out to the legislature the necessary amendments are qualified for the task they have undertaken,—or rather, as it is a much more material consideration, how far the measures proposed by them are likely to answer the ends designed, or are worthy of being embodied into our legal code.

The arrangement of a medical profession on principles which shall ensure to the public a sufficiency of duly qualified practitioners, who shall be able to accommodate themselves to the various classes and conditions of the community, so as to afford to each the best assistance that the present state of medical science will admit of, must be deemed by all a matter of grave and weighty importance. In its accomplishment all ranks of society are interested, and the means of attaining it deserve the maturest consideration. To no being in society, indeed, whatever his place in the scale of social order, intellect, or moral feeling, can this subject be a matter of indifference. It affects equally the peasant and the prince, the philosopher and the clown; the sensualist, whose thoughts or affections range not beyond the boundaries of his own gratifications, and the philanthropist, whose bosom glows with the generous anticipation of alleviating human misery. All are equally concerned in the establishing a more perfect and efficient profession of physic, and should equally rejoice in every rational expectation of rescuing the wretched victims of disease from the venal and rapacious grasp of ignorance and quackery.

These observations I have made to show, first, that some such attempt at illustration as that which I am about to enter on, is become actually necessary; and, secondly, that the subject thereof is one of no ordinary interest or importance.

Unwilling, however, to trespass unnecessarily on your pages, I shall wave all further preface, and proceed at once to the object I have chiefly in view; namely, to illustrate, in the first place, as far as I am able, the present condition of the medical profession in all its departments of education and practice; secondly, to deduce from the history of its progress, and the natural tendencies evinced by it, what those essential constituents are of which an efficient and well-qualified profession ought to consist; and lastly, to point out the means most likely to assimilate the former to the latter state, so as, without trenching on existing rights, or affording any just grounds for complaints of individual injustice, to ensure to the public competent medical assistance, and to the professors of the art a reasonable hope of adequate remuneration.

In pursuance of this purpose, I shall now proceed to specify the several kinds of medical practitioners who are to be found at the present day dispersed throughout the British islands, the mere enumeration of whom is calculated to throw some light on the subject I am treating of, and to prepare us in some degree for judging of their relative merits and advantages.

The following list conveys a fair and accurate representation of them, and will afford much interesting matter for reflection.

*Doctors of Physic of Oxford and Cambridge.*—To neither of these universities does any efficient school of physic belong. They confer medical degrees, however; but rather as being arrived at in the regular course of academic discipline, and attained by a certain observance of acts and terms, than as merited by any full or perfect qualifications in the art of curing diseases: yet these graduates possess privileges such as no other medical men enjoy, and are entitled to demand admission as fellows of the London College of Physicians, without undergoing the scrutiny of an examination, to which all other candidates are subjected.

*Doctors of Physic of Edinburgh.*—A university which furnishes a complete course of medical instruction, and whose degree is only obtained by resident study and examination.

*Doctors*

*Doctors of Physic of Glasgow.*—Here, too, a complete school of physic is established; and similar qualifications required for obtaining a degree, as are insisted on at Edinburgh.

*Doctors of Physic of Aberdeen and St. Andrew's.*—These universities possess no competent schools of physic. Their degree is obtained without either resident study or examination, and on the sole ground of private certificates. The means by which these certificates are procured, the extent to which the system has arrived, and the gross venality and shameless corruption which characterise it, shall be the subject of further discussion by and by.

*Doctors of Physic of Dublin.*—This university, like those of Oxford and Cambridge, grants degrees in physic, considered rather as a branch of liberal science, than as a practical art. They originated at a time when no complete school of physic belonged to it; they are issued on the foundation of the university, and are rather to be received as testimonies of regular literary education, than of medical attainments.

It differs, however, from the English universities, in having now attached to it a school of physic complete in all its parts; for the deficiencies of the school of physic, which would have remained unheeded by the heads of the university, whose attention is otherwise directed, have been supplied by the liberality and public spirit of an individual; and to the late Sir Patrick Dunn, is the Dublin university indebted for having endowed several professorships, and obtained an act of parliament by which the whole is incorporated into one school of physic, which now affords a complete system of medical instruction.

On the foundation of this institution, degrees in physic are also granted, which are analogous to those of Edinburgh and Glasgow. They are not confined to the literary students of the university, and they require a prescribed course of study, and previous examination, in order to obtain them.

*Doctors of Physic of Foreign Universities.*

*Surgeons* of each of the Royal Colleges of England, Ireland, and Scotland;—all differently circumstanced with respect to their connection with pharmacy, and the privilege of combining it with their other pursuits.

*The Scottish Surgeons* are examined in pharmacy, and are even required to produce, on examination, specimens of compound medicines prepared by themselves, as proofs of their practical knowledge of this department.

*The English Surgeons* are allowed to combine pharmacy

 with with their more appropriate pursuits; but they are not obliged to prove before the College their pharmaceutical attainments.

*The Irish Surgeons* are altogether prohibited from combining pharmacy with their other practice, the penalty of expulsion from their College being attached to the offence.

On each of these I shall by and by have occasion to offer some further remarks; they are at present noticed only as forming part of the medical community of these kingdoms.

*The Apothecaries of each Kingdom*;—an appendage to the profession, whose original destination was to dispense the prescriptions of the physician, for which a pharmaceutical education abundantly qualified them, but by no means to practise either in physic or surgery, for which they were utterly unprepared, unless it be alleged that these branches are capable of being intuitively acquired, and without opportunities either for study or observation. They have, however, notwithstanding the disadvantages of defective education, been of late years brought forward by the public as general practitioners; and a due attention to this fact, will be found to afford some views of the medical profession which are in direct opposition to the opinions most generally received. The department of pharmacy has never, that I know of, been regularly legitimatised in Scotland, having never grown into so much importance there as to have acquired a separate constitution. Neither have the mere practitioners in pharmacy throughout Scotland deviated from their proper line of business, or assumed the practice of either physic or surgery. And the fact seems to be, that the public necessities, by which alone they could be so called on, did not require them, being already supplied by a much better general practitioner, namely, the surgeon, allowed by the good sense of his corporation to combine pharmacy with his more immediate pursuits, and thus qualify himself for a department which would otherwise have been supplied from a very inferior order.

Both in England and Ireland pharmacy has been placed under the superintendance of distinct corporations, and principally by reason of the importance attached to this body in consequence of their having insensibly become elevated to the rank of medical practitioners. It does not appear, however, that their chartered rights extend beyond the department of pharmacy, nor have they heretofore so far presumed on their popularity, as to make any attempts at legalising their medical or surgical practice. An endeavor of this kind, however, seems to form a very prominent feature of their intended bill; and it is evident that they now entertain very sanguine sanguine hopes of being able, by making common cause with the surgeon-apothecaries, to establish themselves in the possession of certain legal rights which neither physic nor surgery have ever enjoyed.

The corporations of England and Ireland are differently constituted, and are invested with very different degrees of authority: the English company having no power to control the practice of pharmacy by men not of their body; while the Irish charter empowers the body incorporated thereby, to prevent the practice of pharmacy, even by surgeons and physicians.

The remaining medical practitioners may be disposed of by a brief notice; they are

The *Apothecaries*—not attached to any corporation, but nevertheless largely engaged in the practice both of physic and surgery.

The *Druggists*—dispensing medicines, and also prescribing; and, finally,

The *Grocers*—first commencing by selling drugs by retail, next dispensing prescriptions, then practising the minor operations of surgery, and also prescribing; and, finally, retiring from business with an independence acquired in the course of a very few years, and not unfrequently aspiring to the elevation of a medical degree. And this course, with the exception perhaps of the first step, gives pretty accurately the progress of a large number of those to whom Aberdeen or St. Andrew's degrees in physic are objects of ambition.

Such is the present state of the medical profession in these kingdoms, and of such various and ill-assorted members does it consist. It cannot be that so many varieties are necessary for so simple a purpose as supplying the community with suitable assistance when visited by diseases; and it naturally becomes our next object to inquire which of these are indispensably necessary, and which are to be considered as unqualified, superfluous, or intrusive. To this end it is necessary, first, to take a brief historical survey of the rise and progress of each department, and to show both the order in which they have succeeded to each other, and the causes to which such successive advancement was most probably attributable.

The first medical practitioners recognised by the state, were the physicians. They prescribed for diseases, and the apothecaries dispensed their prescriptions. These latter were, no doubt, soon taught "by doctors' bills to play the doctor's part," and prescribed, in their turn, for those inferior ranks who could not afford the doctor's fee, and who were were consequently relinquished to them without jealousy or reluctance.

The first serious encroachments on the practice of the physician took place when surgery became advanced to its just rank as a liberal profession. Surgeons were then consulted in medical diseases, and this union of both professions in the same individual, giving them a decided advantage over their competitors in public favor, they consequently engrossed a large share of medical practice. And the principle hereby inculcated is still further confirmed and supported by the history of the surgeons themselves; for in exact proportion as they have yielded to or disregarded it, has their own establishment in general practice been confirmed or shaken. This is particularly conspicuous in the history of the Irish College of Surgeons, the members of which complain more of the encroachments of the apothecaries, than perhaps any other surgeons. And with reason, I believe, as to the fact; for this clearly is, that the establishment of a Royal College of Surgeons in Ireland has been followed rather by an increase than a diminution in the number of practising apothecaries. The reverse of this holds true with regard to the English surgeons; for, since they have begun to supply the public with competent general practitioners, combining in their own persons the several departments of physic, surgery, midwifery, and pharmacy, the race of practising apothecaries has been manifestly declining, and will, I doubt not, in due time, become extinct.

The Scottish surgeons seem to have so clearly discerned the nature of the public necessities, and to have so effectually provided for them, that with them the practising apothecary is unknown; and the surgeons and physicians, by coalescing and combining in various ways the several departments, either by superadding them to each other in the same person, or by co-partnerships in business, have supplied their country with medical and surgical aid, such as cannot be objected to.

It is much to be lamented, that the Dublin College of Surgeons have not seen the propriety of emulating the good sense and sound policy of the other surgical corporations, but have allowed themselves to be influenced by vain fancies respecting an imaginary necessity for preserving a perfect distinctness between surgery and pharmacy, in practice at least; that they have indulged in senseless aspirations after an ideal elevation, or equality of rank with the physician, whereby they have been led into the error of opposing the union of pharmacy with their own profession, and have thus directly opened a door for the further intrusion of those who did not disdain to combine every branch in their own persons; they have thus given an impulse to the apothecaries, which has promoted their advancement beyond what any other means could have effected. These two were gradually becoming better educated, and more conversant with general practice; they were consequently more confided in, and were entrusted with the exclusive care of all slighter ailments; while by preceding the surgeon and physician in the treatment of the more formidable diseases, they became associated with them, as it were, in the attendance, though still at a respectful distance, and thus had their information and acquaintance with diseases still further increased, and, in short, became themselves much better practitioners. They still, however, professed only the business of dispensing medicines, and all the corporate rights they acquired were limited to this one occupation. They were not yet, even in the vague opinions of the public, set down as legitimate practitioners, neither did they arrogate to themselves any right of being so considered.

Throughout the whole of this progress we may clearly trace one particular tendency uniformly operating, namely, that of the public to prefer the practitioner in whom the several departments were most completely combined; a tendency so strong, as to counteract the disadvantages of defective education in these latter, and to give the apparent inconsistency of those being most employed in the practice of physic, who were least qualified for undertaking it.

The first attempt to profit by this experience, and to form a practitioner who should unite in his own person every department with a fair presumption that he was duly qualified in all, was on the part of the London College of Surgeons; and in giving to the country the surgeon-apothecary, familiarised to general practice by *apprenticeship*, grounded in a knowledge of the human constitution, and of diseases in general, by attendance on lectures, dissections, and hospital practice, and proved as to his actual attainments by examination, they have done more extensive and essential service to the community than all the other medical corporations united. Perhaps I should associate with them in this praise the Scottish College of Surgeons also, who seem to have been influenced by the same liberal and enlightened policy, and who may even have preceded therein the London College, although my information on the subject does not allow me to pronounce determinately on this point.

It is in contemplating the rise and progress, the excellence and efficiency of this practitioner, the surgeon-apothecary, that that we shall acquire the most correct ideas of what the natural tendencies of the medical profession incline to; and the observations thus made, and the inferences they give rise to, will with considerable certainty instruct us in the best means for making the medical profession what it should be, and for rendering it at once useful and respectable.

Let us now review once more the several departments of the profession, and investigate a little more closely the peculiar circumstances attendant upon each, in order to ascertain, as far as is practicable, the real utility of each, the necessity for its continuance, and the natural extent in which this necessity is likely to maintain it.

The physician, however highly qualified or groundedly learned, it is obvious, can never be considered as a practitioner suited for general practice. In every competition with the inferior classes, he is sure to give way, being superseded by the surgeon, who combines medical with surgical practice, and still more effectually by the general practitioner, in whom every branch is united. It is perfectly immaterial, therefore, what his real qualifications are, how great his superiority, or how vast his merits. He is not calculated for standing in competition with those whom the public, urged by an irresistible impulse, seems determined to prefer, and must therefore yield to a necessity which may be deplored, but cannot be resisted.

The effect of this, however, on the profession of physic, would have been productive of but little evil, if other causes did not concur to degrade and overwhelm this ill-fated department. Were supply of physicians derived from any single source, or even from several sources agreeing in their great leading characters, the only consequence of this declining popularity of the physicians, would have been a decrease of their numbers; they would still have retained the first place in rank and consequence, and finding in due time their natural level as to numbers, would have continued respected and respectable, without suffering individually those neglects and privations, by which such numbers of highly-accomplished and meritorious members of this body have of late years been consigned to penury and obscurity.

The causes of this manifest degradation, and of the severe and trying hardships to which it has given rise, it is almost fruitless now to unfold; for, as far at least as regards the present race of sufferers, they hardly admit of remedy. Some eventual good may, however, attend the developement of them; and at all events the cause of truth is likely to be benefited by such inquiry. May they not be distinctly traced to the numerous and discordant sources from whence the the physicians have been derived—to the want of suitable medical education being afforded by those seminaries, to whose graduates notwithstanding the greatest privileges are by law attached—and, finally, to the corrupt practice of other universities, in granting degrees on private testimonials of qualification only, which testimonials are too often obtained by private favor or gross venality, and granted to individuals whom no university ought consistently with a due regard to its own reputation to acknowledge?

The excess to which this latter system of doctor-making is carried, or the systematic corruption by which certificates are obtained, is far from being generally imagined; and I speak from certain knowledge of the facts, when I allege, that whoever can offer a colorable pretext for making an application to Aberdeen or St. Andrew's, need not be debarred from his degree by any difficulty in finding physicians ready and willing to grant the necessary certificates, provided he is prepared to make the pecuniary recompense required. And the trade seems to be both prosperous and lucrative, for a recently-made doctor of this class, whose university fees at St. Andrew's amounted only to twenty-four pounds, had his total expenses increased by such contingencies as I am alluding to, to one hundred and fifty.

It is of late only that such gross venality and infamous corruption in the profession of physic have come to my knowledge. I have oftentimes, indeed, heard such allegations advanced, but contemned them as groundless calumnies. I have now, however, had but too good proof for asserting, that the granting of certificates to candidates for Aberdeen or St. Andrew's degrees in physic, is become a matter of regular barter and trade; and that a sufficiency of money is all that is required to advance any who has ever dabbled in medicine to the disgraced and unenviable rank of M.D.

I have always been aware that such certificates were too often the compulsory tribute of the needy physician to the more popular apothecary, whose favor he found it essential to cultivate, and whose resentments he dreaded, rather than the freewill offering and unbiassed declaration of an independent mind. And I have even had occasion to know instances wherein men the most honorable and upright, have in unguarded hours been betrayed or seduced into granting certificates of qualification which were afterwards made the grounds of application for medical degrees. But until the fact of such certificates being granted by physicians to men of whom they could know nothing, and to whose persons they were strangers, until brought together in the course of such negociation, had flashed conviction on my mind, of the reality of such enormous conduct, I could not have believed that such practices had any existence.

It may here be reasonably asked, if the profession of physic is so unproductive and ineligible, why is admission so sedulously sought for into it. I merely allege the fact, and confess myself not at all anxious to divine the motives; they lie on the surface, and will readily present themselves to those who may think them worth seeking for.

Who are they who thus seek to dignify their calling, by the assumption of medical honors? Men who have risen from the lowest ranks of medical practice, who have commenced with sweeping the shop and carrying out phials, or even with dealing out groceries by retail, and who progressively availing themselves of the facility by which each advance can be effected, have gone on successively through the several gradations of dispensing and prescribing, until a beneficial treaty for their lucrative business with some junior in the trade releases them from the drudgery which they now learn to contemn, and makes a medical degree the only attainable sanction under which they can continue to levy contributions on the public.

I would by no means be understood as implying that every member introduced to the profession by such degree is necessarily of this character; I know well, that as many of the brightest ornaments of our senate have issued from the rotten borough, so has the profession of physic been occasionally indebted to our rotten universities for introducing into it men of sterling talents and undoubted acquirements. But no amount of such advantages can possibly compensate for the great and glaring evil of throwing open the profession to such incompetent pretenders as I have been describing; nor can the profession itself do otherwise than descend in the scale both of usefulness and respectability, so long as this much-abused privilege is enjoyed and exercised by these prostituted bodies.

The evil, too, is almost without counterbalance; for, were these creditable practitioners who are occasionally supplied with degrees from Aberdeen or St. Andrew's, even more numerous and valuable than they really are, it is not to be overlooked, that the claim of the universities to the merit of bringing forward their talents or abilities, stands low indeed. Their services were given fully to the public before such medical degrees were obtained; and here the analogy with our senate totally fails: for, whatever force such an argument may possess in defence of a corrupt system of representation, it can assuredly have none when applied to the corrupt manufacture of physicians. What, then, is the benefit derived from granting medical degrees on private certificates only? Does it extend a single point beyond the idle gratification of the individual, the indulgence of a vain pride, if not of some more hateful feeling, the pecuniary advantage of the certifying physicians, and the payment of certain insignificant fees to the funds of the universities? And are such considerations worthy for a moment of being deemed a sufficient excuse for continuing a system by which a liberal and truly-dignified profession is so abused and degraded?

A further cause of the degradation of physic will be found in the ill-judged proceedings of those bodies who have existed only for the purpose of protecting and advancing it; for, judging by false principles, and overlooking the uncontrolable tendencies of the public mind, they have almost uniformly by their procedures increased the difficulties of regular admission to their own body, have required harassing and vexatious examinations of men whose qualifications had already been duly attested after sufficient ordeals, have called for pecuniary fines, oppressive in their operation, and not required for nor appropriated to any single valuable purpose; in fine, have tended most powerfully, by diminishing the number of regularly-educated practitioners in physic, to leave a vacuum which the unqualified were sure to supply. These general reflections require that further facts should be adduced in support of them; and the most forcible and impressive way of bringing such forward, will be to present them in contrast with the corresponding facts derived from the history of other medical corporations.

Let us compare, for instance, the conduct of the two medical corporations of the metropolis, in some of the most essential articles of their internal government. The College of Physicians of London examine candidates for country practice, who may or may not be graduate physicians; and they elect them extra licentiates of the Royal College.

It may be observed by the way, that, according to high legal authority, their legal control does not in reality extend to the situations for which they affect to license these practitioners; nor do they confer on such any privileges by their license, beyond what was previously possessed. Having no power then to protect such practitioners in any respect from the encroachments of irregulars, it may fairly be asked to what end do the graduates of regular universities, whose degrees have been obtained after resident study and examination, submit to a further examination, and the exaction of fees by this body? For myself I answer, that I can see no one sufficient motive for such compliances; and that I conceive the extra license of this College to be to all intents and purposes utterly valueless.

But to the licentiates of the College they grant a different license, empowering such to practise in the city of London, and within seven miles around, being the limits to which their jurisdiction really extends, on their undergoing a different and more strict examination, and on paying other and more considerable fees. Now to this distinction I most decidedly object, as not being defensible upon any sound principle. Why practitioners should be required for town practice, superior to those to whom the country population is committed, I own I do not fully comprehend. The idea is by far too courtly for my rusticated mind to entertain or imagine; nor can I, for this greater caution respecting town practitioners, and more rigid examination of them, discern any motive, but a regard to private interests, a jealousy of encroachment, and a disposition to limit the number of competitors.

In all these respects, the conduct of the London College of Surgeons may be contrasted with that of the Physicians, much to the credit of the former. The surgeons examine generally, and qualify with their fullest license any young man who has undergone the necessary *apprenticeship, and attended the necessary lectures, dissections, and hospital practice.* If destined to practise in the country or provincial towns, his fees are reduced on the principles that he thus foregoes those peculiar advantages which closer attachment to the corporate body, and eligibility to its offices, would in town practice impart to him. They leave him too at liberty to practise pharmacy, and thus enable him to enter into successful competition with those practitioners by whom in country life he is most likely to be opposed.

Should a practitioner of this class wish to advance his fortunes in city practice, and be induced by a laudable ambition, or any other motive, to adventure therein, he meets with no mean or pitiful jealousies to oppose his intentions, but is at once received without further trials, and is only required to pay to the funds of his College his additional fees; a measure perfectly equitable and proper, and which is fully justified by the obvious propriety of his contributing more largely to the maintenance of that establishment by which he is so likely to benefit, and to whose offices he may be elected, than when the benefits imparted to him were less sensibly experienced, and the election to office impracticable.

It ought further to be mentioned, that a bye-law of the College excludes from its offices all such members as are actually actually engaged in the practice of pharmacy; but, with a liberality which cannot be too much commended or admired, they have abstained from rendering the law retrospective, nor can it affect any member who, though he may have practised pharmacy, has relinquished it in order to attain to official distinction in his corporation. These traits of liberal sentiment and sound policy in the constitution and government of this admirable institution, entitle it in my mind to the highest respect; and if we regard the extensive advantages that have resulted to society from the establishment of that excellent species of practitioner the *surgeon-apothecary*, and reflect on the short period within which the activity of this College has been so beneficially employed in supplying to the public what they stood so much in need of, we can hardly, I think, rate the merits of this body too highly.

It would be invidious to compare them further with the College of Physicians, or to dwell longer on the less judicious course which this latter body has thought proper to pursue. That they have done so with pure and upright intentions, though on mistaken principles, I am willing to allow; nor do I here expose the shallowness of their policy, or the injurious tendency of their procedures, with any other view than to lead to their correction. What powers should be exercised by, and what authority invested in, this body, I shall by and by have occasion to treat of: at present it is necessary for me to proceed in the review of the several classes and corporations which remain to be noticed.

Previous to the supply of practitioners for general practice by the College of Surgeons, according to the wise and efficient system on which they have acted, the apothecaries were in the exclusive possession of this extensive field; and, however ignorant and unqualified they originally were for undertaking its duties, there can be no doubt but that they have had diligence and good sense enough to supply many of their deficiencies by the assistance of the private teachers and public hospitals of the metropolis, and that they now remain a numerous and respectable body. Among them may, I doubt not, be found many individuals possessed both of learning and abilities, and who, by their association, would reflect credit on any College, whether of Surgeons or Physicians; but while I acknowledge the services they have rendered to society, and willingly accord to them the merit of attaining considerable improvement, when under no obligation to effect this, save what moral principle and a laudable ambition imposed on them, we must not lose sight of the imperfections of the system which elevated them into practitioners, practitioners, nor be blinded to its utter incompetency for ensuring to the public the due qualification of each individual candidate for practice who may come forward under its auspices.

No degree of individual merit can possibly atone for the general inferiority of this class of practitioners to that of the *surgeon-apothecaries;* nor should any false estimate of their supposed equality mislead us for a moment into sanctioning any system which should tend to give perpetuity to this body, by legalising their practice, or encouraging their increase. If left to the natural influence of time and of causes continually though silently operating, they will, I doubt not, in due course give way to their better qualified rivals; a change which has to a considerable degree already taken place, which is still in full progress, and which, if not interrupted by injudicious legislation, will assuredly establish the surgeon-apothecary eventually as the general practitioner all over the kingdom; and it will accomplish this without occasioning a single instance of individual injustice, or in any respect oppressing that body whom it is intended to displace. How such salutary change may be best promoted, so as neither to affect individual interests, nor hazard the retardation of the natural progress of events, must be further discussed when we come ultimately to speak of those amendments of the profession in which the aid of the legislature may be beneficially employed.

I come next to notice the medical corporations of each of our other metropolises; and it is the more necessary to display the peculiar attributes of each, as a knowledge thereof must greatly tend to facilitate that free and reciprocal intercourse which ought assuredly to exist between the several parts of the same empire.

The university of Dublin, as I before observed, grants degrees in physic on two foundations: the one that of their charter, which having been granted at a time when medical science was but in its infancy, does not make provision for the due information of the candidate. It is accessible only to the literary graduates or under-graduates of the university, or to those who may be admitted to degrees *ad eundem* in Trinity College, Dublin, by a *bene decepit* from Oxford or Cambridge. They afford no sufficient proof, however, of a competent medical education or acquirements, and they do not, that I know of, confer any privileges beyond the precincts of the university.

The other medical degree which issues from Trinity College, Dublin, is attainable by candidates who are not literary graduates. It is conferred under the authority of an act of

1 parliament, parliament, by which Sir Patrick Dunn, himself a medical practitioner, was empowered to found, in the university of Dublin, a more perfect school of physic, and to endow out of his private property certain professorships that were previously deficient.

To the three medical professorships of the university he added three others, so as to complete the six of which all our principal schools of physic now almost uniformly consist. A course of study is required by this bill nearly similar to that which obtains in Edinburgh and Glasgow: a thesis is published, examinations held, and a degree granted, which, as far as any such instrument can convey the assurance, testifies to the public that the person possessing it has at least gone through a regular medical education. And while I am on this subject, it may not be amiss to observe that the language of a medical degree, the qualifications it vouches for, and the privileges it confers, have been made the subjects of much unmeaning ridicule, and their declarations egregiously misconceived, in consequence of a too literal acceptation of them.

The true extent and import of this instrument are so well explained by the Senatus Academicus of Edinburgh, in the reply given by them to Dr. Harrison in 1807, that I shall make no apology for introducing the extract in this place. The committee appointed by the Senatus Academicus to consider and report on Dr. Harrison's proposals, after some observations on the nature and tendency thereof, proceed to remark, that "they need not mention the origin, and the reasons of conferring degrees in physic, a practice which has been long followed in Europe, and of which the expediency and even necessity are now universally acknowledged. They beg leave, however, to observe, that it has never been intended to declare to the public, that the person upon whom a university confers a degree of Doctor of Physic, has acquired a complete knowledge of medicine; but to announce to the world in the most public manner, that he has been regularly educated, that he has studied during a university period all the branches of medicine, and that he has acquired such a stock of knowledge as in the opinion of competent judges qualifies him for entering upon the practice of physic."

After such an open and candid declaration, neither the quaintness nor turgescence of expression which may appear on the face of a medical degree, can reasonably subject it to either ridicule or reproach. It is possible that a more simple form of words might be devised, more accurately expressive of the degree of approbation due to the candidate; but it does does not appear that the evil of the present form is of such magnitude to call for innovation in respect of it. The subject indeed is little worth discussing; but, as among other sarcastic and disingenuous reflections cast on this body by their rivals and opponents, with a view to depress them still lower by rendering them the objects of ridicule and contempt, this one has been not unfrequently resorted to, and with some effect on the light-minded and inconsiderate, I thought it not unmeet to place the matter in its proper light, and thus to vindicate the possessors of such degrees from the imputation of an arrogance which they are utter strangers to, and cannot feel.

The Dublin College of Physicians examines for licenses to practice in Dublin, any medical graduate of a university. Unlike the London College, however, which is obliged to admit to its association, without further trial, the doctors of Oxford and Cambridge, the College of Physicians of Dublin recognises no peculiar claim in the medical graduates of their own country, but examines indiscriminately all who come forward to require their license. They further demand the sum of fifty pounds as a fine on admission, to which amount it has been raised within a few years from five-and-twenty, not from any wants of the College for an extension of their funds, but on the alleged principle of increasing, by such increased expenditure of the candidate, the respectability of the profession. By what mode of inference such a conclusion was arrived at, or by what species of logic the reasonings which led to it were conducted or supported, I own myself too dull in intellect to be able to discover.

This College has never been called on to examine country practitioners, nor do I believe the expedient of granting extra licenses has yet occurred to them. There are some further circumstances, however, respecting this body, so illustrative of the general interests of the profession of physic, and so expressive of the spirit with which corporate powers are so prone to be exercised, that it cannot be a waste of time to expose these a little further.

The management of this corporation rests solely with the fellows, and does not extend to the licentiates. These fellows elect from among the licentiates as they think fit. Conceiving themselves, as they naturally did, to be placed at the head of their profession, and invested with such power of limiting their numbers, it was hardly to be expected that they would have the virtue to withstand the temptations to selfish and sinister proceedings thus offered to them, nor that they should not lend an ear to that specious but delusive reasoning, which whispered to them, that as holding the the highest rank in the profession they must consequently be the most esteemed of medical practitioners, and that the amount of business and emoluments engrossed by them, was very likely to be in the inverse ratio of their numbers.

How far this affords a correct elucidation of their conduct in this particular, I shall not pretend to aver; but the fact is certain, that when Dr. Harrison first called the attention of the profession to its manifold grievances and abuses, the fellows of this body resident in Dublin, and exercising all its rights and privileges, were reduced to ten only.

At this period the licentiates of the College were between thirty and forty in number, and might constitute about one half of the practising physicians of the city, the remaining part consisting of graduate physicians of different universities, who for divers reasons had not become attached in any way to the College, but continued to practise independent of its sanction. And it is not a little curious to develope the motives by which so large a body of practitioners were withheld from incorporating themselves with the College by obtaining their license. Some of high minds felt averse to submitting themselves to the overbearing spirit by which this little conclave was obviously influenced; others dreaded the power which might be exercised over them at examinations by men whose obvious policy it was to narrow the profession, and exclude from its pale; others again were either unwilling or unable to pay the advanced fine of fifty pounds, more especially as they knew its destination was to be expended not on objects of science, but in drinking and carousing. The latter of these, indeed, or those who pleaded inability, the College most liberally offered to compromise with, by agreeing to accept half their demand in cash, and the remainder in approved bills at twelve months date. But I have not been able to learn that this generous tender was received with the gratitude it was so justly entitled to.

It is further worthy of remark, that although this College were empowered by their charter to fine all such physicians as should be found practising in the city without their license, these unlicensed men whom I have been noticing were left unmolested, and suffered to practise on an equal footing with the licentiates, who had yet been obliged to purchase their privilege by undergoing an examination, and paying the sum of fifty pounds.

Shortly after the period I have been adverting to, the unlicensed physicians of Dublin entered into an association with certain of the licentiates, for the purpose of procuring some amelioration in the management of the affairs of the College,

College, with regard to the examinations for licenses, and the fines demanded on obtaining them. Unsupported, however, in their endeavors, and despondent of success, this association, after several communications with the College, in which nothing definitive was determined on, became insensibly dissolved, some members being admitted to licenses unexamined, others consenting to undergo examination, while several still maintained a proud and stubborn independence, holding out equally against the concessions of the College as against their more rigid demands.

The measure of association, however, has not been wholly unproductive of good, for since that period the College has considerably increased the number of its fellows, but without entering into any regulations for altering the mode of electing them, or providing any security against the effects of a monopolising spirit, whenever this shall again spring up among them.

In the Dublin College of Surgeons, though I find somewhat to condemn, yet I have infinitely more to approve of than in the sister corporation. And it is interesting to observe, that both here and in London, the institution of recent establishment evinces much more both of liberal sentiment and sound judgment, than that which claims so much higher an antiquity.

This College examines for surgical licenses all who have undergone the *necessary apprenticeship*, and attended the necessary lectures and dissections. The examination for a license is their principal one. They examine, however, both surgeons and mates for the army and navy, and supply to both a large number of medical practitioners.

The licentiate, in two years from his examination, is eligible as a member of the College; and unless he is chargeable with some instance of medical or moral delinquency, he is elected on a simple application, and admitted on paying a further inconsiderable fee.

To this body is Ireland indebted for furnishing its population with an abundance of surgical practitioners, well grounded in anatomy, and the knowledge of surgical diseases. The errors of this establishment are, that affecting an equal elevation to that of the physician, and endeavoring to preserve between the several departments of physic a forced and unnatural distinctness, they have disdained too much the conjunction of pharmacy with the practice of their art, and on most mistaken principles have prohibited their licentiates therefrom on pain of expulsion.

And indeed the combination of pharmacy with surgery in the person of the surgeon, seems in Ireland doubly guarded against, and utterly impracticable; for should the surgeon, urged by his own necessities, and the pressing wants of a provincial district, resolve on foregoing his license in preference for what would much more materially serve his immediate interests, he would soon find the establishment of a dispensary opposed by another chartered body, the company of apothecaries, who would prohibit his practice in this line unless he were to become a member of their body, a matter by the way utterly unattainable by such practitioner, inasmuch as it requires a long *apprenticeship to pharmacy*, as well as examination, in order to accomplish it. Thus, in their zeal to maintain the dignity of surgery, and to hold an equal rank in society with the physicians, they have overlooked altogether the wants of the public at large for a general practitioner, and have actually imposed on the apothecaries a positive necessity for assuming the character, with what advantages to the community I need not descant on. That these have supplied the want in the best way that the case admitted of, I readily grant, and if any reproach attaches to their manifold incapacities, I am far from imputing an atom of the blame thereof to the apothecaries. They only fulfilled the wishes of the public in becoming general practitioners; and if this class has in Ireland been exclusively formed by superadding to pharmacy the practice of physic and surgery, without any qualification in these latter departments being ensured or attested, instead of the more rational mode adopted by the London College of Surgeons, of leaving the practice of pharmacy open to the well-informed surgeons whenever the nature of the population rendered a union of the departments necessary, the blame must rest any where rather than with the apothecaries. They are now a respectable and deserving body of men, and may well feel some indignation at the supercilious treatment of their more dignified competitors.

But while I appreciate their merits, and vindicate their character, I by no means intend to offer a single plea for this department of medical practice being resigned to them. On the contrary, I conceive it a matter of actual necessity that it be transferred to other and more competent hands; and if the Dublin College of Surgeons will only emulate the liberality and sound policy of the London, and empower their licentiates to combine pharmacy whenever the wants of society render it expedient, the control of the Apothecaries Company being at the same time removed, there can be no doubt but that this change would take place by a silent and unperceived, though certain and progressive operation, without any individual injustice being exercised, or any interruption taking place in the necessary attendance on the public. As one body of practitioners increases, the other will insensibly decline. Young men entering the profession will naturally qualify themselves for that line which is likely to prove both most lucrative and most respectable, and thus a body of practitioners will in due time arise, for each and every one of whom the public will have a full assurance of regular education, and perfect qualification.

Were such the condition of the general practitioners, we should soon cease to hear of their murmurings or complaints at receiving inadequate remuneration; complaints which proceed from the excessive numbers and disorganised state of the profession at large, which has engendered a most disgraceful species of competition among the rival practitioners, and by inducing the worst among them to advance themselves by underselling their opponents, establishes them on the ruins of the more meritorious members, and finally vilifies and degrades the whole.

I have dwelt long on this subject, both in this place, and when treating of the London College of Surgeons; and I do so the more readily, because I conceive it to be the point on which the establishment of a competent body of general practitioners must depend. It is one too on which much prejudice prevails, even among enlightened men; and on this account I am the more anxious that its real merits should be thoroughly understood, and that the sources of fallacy which have heretofore obscured the reasonings, and clouded the judgments of those who have considered it, should be detected and exposed.

The two great obstacles to the arrangement which I advocate, are, an impression on the minds of surgeons that their dignity would be compromised by allowing their members to conjoin pharmacy with surgery in their practice; and a sort of indistinct and unsettled notion in the minds of the reflecting part of the public, that such combination must necessarily engender in the mind of such mixed practitioner a constant struggle between self-interest and moral principle.

On both these heads I shall now offer a few remarks, and then dismiss the subject altogether.

That the dignity or real respectability of any profession shall be lessened by that which materially increases its usefulness, is a position which will not be easily maintained; and, though that of the individual members who hold the highest stations might suffer were they to continue to dispense medicines when so elevated, it does not appear consistent either with reason or fact to conceive that these can be affected in any any such way by the dispensing of medicines by the subordinate members; and, as I can show pretty clearly, by reference to facts, that this consequence does not result from such combination, I shall be satisfied to try the question by this test alone, as being that concerning which there can be the least cavilling or disputation.

The London College of Surgeons grant their highest license to men who practise pharmacy, yet it does not appear that any disrepute in consequence attaches at the present day to the names of Cline, or Cooper, or Abernethy, or any of the almost endless train of scientific and enlightened practitioners whom it has the honor of claiming as members of its body. I appeal to general experience too, whether the independant surgeons who are to be met with in all the larger towns of England are not to the full as respected, and respectable, as any members of the Irish College. Nay I may carry my argument still further, and show that even the dispensing of medicine by the individuals themselves is no bar to their arriving at high eminence, or attaining the first respectability; and, in proof of the position, I may confidently appeal to our third metropolis, in which all the very highest members of the profession of surgery keep their own shops and dispense their own medicines; yet it does not appear that the names of Bell, of Russel, of Wardrop, of Wood, or of Thomson, or any of the enlightened and liberal practitioners in surgery with which the city of Edinburgh abounds, are either the less respected by the public, or the less esteemed in private society. It is surely unnecessary to go beyond these proofs, or to advert to the almost universal practice of the American physicians, with whom, if I mistake not, it is even a matter of legal injunction to combine pharmacy with the practice of physic.

I trust I have sufficiently shown that the wants of society clearly require such combination; that it is infinitely preferable to effect this by conjoining pharmacy with surgery and physic, rather than by suffering these branches to be assumed without proof of qualification, by the mere practitioner in pharmacy; and finally, that it does not derogate from the real dignity of a Royal College, or impair the respectability of its members, to admit of and authorise such combination.

To argue at length the question whether the science of medicine has been benefited by the subdivisions of its practice, and whether and to what extent it would be desirable or practicable to re-unite them, would lead me far beyond my present purpose. The position that such subdivision has conduced either to its improvement as a science, or to its beneficial application in practice, is one which I am convinced may be satisfactorily refuted.

It is not many years since this question became the subject of a prize essay in a German academy, and produced fourteen answers, of which thirteen were in favor of the reunion and one against it; yet so inveterate are long-established prejudices, and so ineffectual the light of reason in dispelling the mists with which their objects become enveloped, that to this one the prize was adjudged, although in every page the author is seen to struggle against the suggestions of his better reason, and is finally found to negative, not the direct question of the academy, but a peculiar modification of it by himself. I need hardly refer my readers to the Edinburgh Medical Journal for January 1807, for a further account of this interesting discussion, as the book must be in all their hands, and the paper I allude to familiarly known to them.

Of the apothecaries of Ireland I need say but little. They are in part the general practitioners of the country; but the surgeons, who are become numerous, successfully dispute this ground with them, and notwithstanding the disadvantage of not being allowed to combine pharmacy with their own profession, they yet are very extensively employed in the practice of physic, and hold as it were a middle place between the physicians and apothecaries. Still these latter engross a very large proportion of both medical and surgical practice, and their encroachments have been the subjects of loud and lasting complaints by both surgeons and physicians.

In short, so strangely perverted and unharmonised has the whole medical profession become in this country, that it is impossible to conceive any change that could be productive of equal recrimination. The surgeon exclaims against the apothecary, the physician accuses both, the apothecary retorts, and thus they go on mutually exasperating each other by every vilifying epithet and opprobrious insinuation, until they have rendered life such a scene of heart-burning animosity and contention, that the strongest feeling of every liberal mind must be a desire to escape for ever from the profession and its bickerings, and to seek some Lethean balm by which his wounded spirit may be healed, and rendered oblivious of all the harassing and depressing cares with which professional life is so thickly beset.

The apothecaries have a corporation which superintends the education and practice of pharmacy, its control extending all over the kingdom. It affords no assurance, however, either of medical or surgical attainments on the part of those examined, examined, although they are necessarily destined to practise extensively in both departments. This corporation is also a joint stock company, and divides a large per centage, from the profits of drugs sold at the Apothecaries' Hall.

I shall now briefly advert to the state of medical education and practice in Scotland, and then conclude with a few observations relative to those measures which would tend most to improve the general condition of the profession, and which should, consequently, form the basis of any legislative enactments that might be intended to accomplish this desirable purpose.

The university of Edinburgh provides a complete course of medical instruction, and confers degrees in physic after resident study and examination.

That of Glasgow does the same.

The universities of Aberdeen and St. Andrew's possess no schools of physic, but they confer degrees in physic, notwithstanding, on those who bring certain certificates from individual practitioners, and who pay the fees, amounting to twenty-four pounds. Such is the present price, as officially notified by a letter from St. Andrew's, now before me, dated in the present year; but it seems to have been enhanced of late years, for, unless I am much mistaken, the cost of such degree was formerly but thirteen pounds.

The Edinburgh College of Physicians are obliged to admit all doctors of the Scottish universities to their association without examination, and only on paying the necessary fees. They may examine graduates from England, Ireland, or foreign universities; but I rather think their practice is to be satisfied with inquiring into the education and previous qualifications of such candidates, and that, finding them to have been regular and complete, they grant their license without further difficulty.

The surgeons of Edinburgh examine their candidates both in surgery and pharmacy, and they even require specimens of compound medicines prepared by the candidate, to be exhibited in proof of his practical acquaintance with the art. How far they inquire into his medical attainments, I am not prepared to say. They thus provide a general practitioner equally proved as to his qualifications for general practice as is the *surgeon-apothecary of England*, and equally combining in his own person the several departments. From this source the general practitioners of Scotland seem to be principally derived, nor do I find that mere apothecaries prevail among them. They are certainly not sustained by any corporate rights, nor do they seem to claim any particular attention.

The

The surgeons, as has been already observed, are allowed to combine pharmacy without any limitations or restrictions, and accordingly the very first surgeons of the city dispense medicines for their own patients.

The question has, some time back, been warmly contested in the Edinburgh College of Physicians, whether the members thereof should not also be permitted to dispense medicines, and the bye-law which prohibited them from doing so be rescinded. The controversy it gave rise to must be too fresh in the minds of my readers to render it necessary for me to do more than merely advert to it in this place. The proposition for repealing the law was numerously and respectably supported, but it was opposed by the distinguished professor of the practice of physic with all his characteristic ardour, and, I believe, finally fell to the ground, a fate which I cannot but rejoice at, as I believe the proposition itself to have been founded on very mistaken views of the medical profession; for, though I lay no stress whatever on the objections to the measure, grounded on certain moral principles that were brought against it, for the reason, that, as the combination of departments must take place in some individuals, I conceive that less moral turpitude is likely to attend the practice when committed to the higher and better educated orders of the profession, than when consigned to the lower, still I cannot see adequate reasons for admitting such combination to take place in the persons of the physicians. The strongest motives which justify such combination in the surgeons and general practitioners are here wanting, for even with this assistance the physician cannot become qualified for general practice. Surgery would still be wanting to him, without which he must be utterly incapable of undertaking the duties of this line of practice.

Much better is it then to leave it altogether to those who are most competent to undertake it, and that the physician should appear only where his services are required, and where they can be duly rewarded without such adscititious expedients. Nay, I am not at all sure that such combination in his person would not very effectually lessen his own dignity, and degrade his department. It is the motive, rather than the act, that produces such consequences, and with him it could only proceed from sheer love of gain, without any peculiar advantage resulting to the public.

Having now thoroughly reviewed the actual state of medical practice throughout the British isles, I trust I have succeeded in demonstrating, with some degree of clearness and precision, the natural tendencies which it affects, and the injurious influences which at present pervade it; and, if my arguments arguments in favor of that union which must take place in some order or other of practitioners, and which no restrictions on the part of chartered bodies have power to prevent, are of any weight, the prejudices which have so long contended against this must give way; the misapplication of certain principles taken from writers on political economy, respecting the advantages resulting from a division of labor, become manifested; and many obstacles be removed that might be opposed to these regulations, which would relieve the profession from its greatest grievances, and render it at once valuable to the public, and beneficial to the members composing it.

In the detail of these measures I shall be very brief, and indeed shall do little more than merely hint at them; for though I have investigated, and I trust with some success, the source of many of the evils which affect the profession, I have by no means prepared myself for pronouncing decidedly on those remedial measures by which they should be corrected. If the principles advanced, however, are sound and just, the nature of the remedies required cannot long be a matter of doubt, but must flow naturally from a knowledge of the tendencies to be encouraged, or the evils averted.

It appears pretty clearly, I think, from the foregoing inquiry, that the public must have a practitioner for general purposes, in whom the several departments are more or less combined, and that the advantages of such combination to the practitioner are such as to counterbalance the many great and glaring defects with which practitioners of this class have been so frequently and not undeservedly reproached; and the further result of my observations respecting the combinations of the different departments in the superior classes of practitioners, is this, that the physician may exist without combining with his own profession either surgery or pharmacy; that the addition of the latter would not qualify him for general practice unless surgery and midwifery were also combined; and that the experiment, if tried, would be found utterly to fail in giving to the physician a parity of advantages with either the surgeon or general practitioner.

The surgeon may dispense with pharmacy, and may send his prescriptions to the druggist, who is now in fact what the apothecary was formerly; but he must in self defence combine the practice of physic in considerable proportion, else he must yield his own place to the more general practitioner, and sink into even greater inactivity than the physician, inasmuch as the quantity of medical practice greatly exceeds the surgical; and even in large towns it is well ascertained, certained, that adherence to surgery alone will hardly procure for its votary common subsistence, unless he has already arrived at high eminence in that department.

The candidate for general practice must in his own person combine all the several departments of physic, surgery, midwifery, and pharmacy, if he means to have a fair prospect of succeeding as a popular practitioner. The only question that remains, therefore, respecting this class, is, whether it is adviseable to form them by encouraging the proved licentiates of the surgical colleges to combine pharmacy with their other pursuits (a measure so beneficially resorted to by the Colleges of London and Edinburgh); or to leave the apothecary to engraft on the art of dispensing, the more abstruse ones of surgery and physic, without having his qualifications in these respects at all vouched for or ascertained. That many of the class of apothecaries are as well prepared at the present day for general practice as any of the surgeon-apothecaries, I believe to be a fact; but for such perfect qualification we are only indebted to the industry and moral principle of the individual, and should be lamentably disappointed were we to calculate on these qualities being possessed by every individual of this body, or indeed of any numerous body in existence. Such a supposition would be against all experience, and could never justify our leaving open so important a profession as that of physic to men for whose capabilities we have no assurance but such as their general characters for integrity and moral rectitude may convey.

To the other class of general practitioners there can be no possible objection. They are thoroughly qualified, as far as it is practicable to accomplish this, or rational to expect it; and their qualifications are proved. They are rapidly taking place of the less eligible practitioner throughout every part of the kingdom; and, in truth, the presiding powers have only not to interfere or interrupt a revolution which is actually taking place with sure and certain progression.

It would certainly materially facilitate and expedite this desireable change, and tend still more effectually to render identical the class of general practitioner throughout the kingdom, if some measures were adopted for incorporating with the surgeons the rising generation of apothecaries, by admitting them to examination for licenses from the Colleges, and previously pointing out the course of study that should be required from such candidates. The multiplication of regular practitioners would then proceed with inconceivable rapidity, the public would be supplied with them in proportion to its wants, injurious competitors cease to exist among among men who would then be equally and similarly qualified, and in due time the whole department find its proper level, so as no longer to be overrun by excessive numbers, the great cause of that inadequate remuneration so loudly and generally complained of. To legalise the present race of apothecaries by any system of examination or license, I conceive to be a proposition absurd in the extreme. As all men now enjoying medical practice must be left in undisturbed possession of the same, however ill qualified they may be, let the present race pass away, and be replaced by a better order of men, as they undoubtedly will unless the natural operation of things shall be interrupted and impeded by some such injurious measure as the bill about to be presented to parliament.

In England, the system which is to supply these better-qualified practitioners is in full operation, and will produce its effects independent of any extraneous aid.

In Scotland too the members of the College of Surgeons seem to supply the population of that country with all the general practitioners they require.

But in Ireland an essential change must take place in the conduct and sentiments of the Dublin College of Surgeons, else they need never hope to supersede the apothecaries in the field of general practice, nor to render the class of general practitioners what they ought to be. Let this body lay aside the false pride which forbids its licentiates practising pharmacy; let these accommodate themselves to the state of population in their respective districts as circumstances may require, and either combine pharmacy or not with their other practice, as their necessities or inclinations shall prompt them; and a numerous, respectable, and well-qualified body of practitioners will spring up, whose superiority over the uneducated apothecary will be speedily discerned and acknowledged, and who will gradually and imperceptibly displace these to the decided advantage of the public. The necessity for such a change too in Ireland is the greater, because the apothecary has not there equal opportunity for improving his qualifications as the similar practitioner has in England, the Irish hospitals not being in general so well arranged for affording instruction to pupils as those of London, nor are the private teachers by any means so numerous or so well established.

To all enactments for regulating the price of medical assistance, or legalising its specific demands, I am decidedly hostile. The fluctuating value of money must render it a matter of endless revision, independent of every other sound and weighty objection that may be brought against such

 provision.

provision. That it is necessary, generally speaking, to enforce payment for professional services, I disbelieve; and if the operation of such causes as I have been exploring, has given rise to an injurious competition among medical practitioners, and by introducing among them some of the meanest and basest of mankind, has originated within this body a system of underselling as it were, and of superseding each other by every fraudulent and unworthy art,—the remedy must be sought, I should think, not in giving power to the regular practitioner of enforcing payment of his demands, but in effecting those salutary purifications of his profession, which would render such compulsory measures unnecessary.

Were the experiment tried even, and the fullest power granted by statute to enforce payment for professional services according to any established scale of remuneration, I am thoroughly persuaded that not a single individual of this body would eventually profit by possessing such. A sufficient number would be found to avail themselves of so golden an opportunity for vaunting of their own moderation and liberality, and by declining to exercise an authority so injudiciously accorded to them, would effectually supplant all those who should have the temerity to resort to it. Nay, the very men who are now loudest in their complaints, would, most probably, be among the foremost to apostatize from their own system, and would thankfully accept the voluntary payment even though beneath the regular demand, rather than exact the full dues by legal process. In short, a more injudicious measure, I conceive, could hardly be contemplated; and I am perfectly satisfied that its effects, if carried into a law, would, both on the general profession, and on the individuals composing it, be those of unmixed evil.

I have now disposed of the great and most important question, that of the general practitioners, and I call it so without fear of contradiction, for on the competency of this class must the great mass of the population rely for the preservation of health and removal of diseases. To by far the largest portion of society they are the sole physicians, and even the highest ranks are known to depend with the fullest confidence on their skill and ability. Domesticated, as they so frequently are, in the families of the great, these have continual opportunities for appreciating their merits: they become familiar with their characters, acquire a personal attachment to them, place an entire confidence in their skill and integrity, and finally, when trying circumstances arise, or dangerous diseases assail them, yield with reluctance to the demand for further assistance, and are ultimately ultimately induced to call in the surgeon or physician, more from a desire of indulging their favorite attendant, and skreening his character, than from any hope of benefiting by the compliance.

For these and many other considerations, I shall be pardoned, I trust, for having dwelt so much on this particular subject, and for having given it so prominent a place in the present inquiry. If its merits are once rightly understood, no difficulty can afterwards attend the due regulation of the other departments, each of which will speedily find its proper level, and the whole become harmonised into one perfect and efficient political institution.

Respecting surgery, I have only to lament, that the system of education provided for it does not necessarily include a larger portion of medical knowledge, and that this is not more dwelt on in the course of a surgical examination. I suspect much that the surgeons are restrained from combining these subjects by some false and untenable principles respecting an imaginary necessity for keeping the professions utterly distinct, and by a sense of honor towards the corporations of physicians, and an unwillingness to trespass on their province; but when it is once clearly understood that they have to qualify men not merely for surgery but for general practice, and further, that even those members of their body who profess surgery exclusively, are nevertheless under an absolute necessity of practising physic likewise, it is to be hoped they will see the propriety of correcting their views, and not only promote the acquirement of medical information by making it form a suitable part of the preparatory course of education, but openly and manfully avow their purpose by constituting it a subject of examination.

A few observations now on the department of physic, and I shall conclude.—This body must not aim at furnishing to society general practitioners. They must be satisfied to exist in the greater towns, and more populous districts, where only the separate departments can be severally maintained. And with this they should rest contented, as it affords an ample field and full encouragement for their talents and exertions. The field, however, must not be overstocked with laborers, else the harvest to each must be small and inadequate. All irregular supplies, and especially of incompetent physicians, should be at once cut off. The Scottish universities, which grant degrees on private certificates only, should have the privilege rescinded. The English universities, too, should either establish in each a competent school of physic, or they should insist on a previous course course of study, similar to that required by the other British universities, so as to render their degrees equally conclusive respecting the education and qualifications of the possessors, as are those of Edinburgh, Glasgow, and Dublin. Such harmony and consentaneous proceeding on the part of the universities by which these kingdoms are supplied, would take away all pretence for any subsequent examination by the Colleges of Physicians, whose office would then be wisely restricted to examining into the reality of the degree produced by a candidate, who on due proof of its having been regularly obtained, should be forthwith admitted.

A power should not be vested in these bodies, unless under very particular circumstances, to retry a medical graduate, whose degree had been obtained after resident study and examination; nor should they be subjected to the temptation of throwing those obstacles in the way of such candidates, which a selfish and narrow-minded policy, and a jealousy of encroachment, might suggest to them. A power of this kind they manifestly possess, so long as they retain the right of examining medical graduates; and while this is the case, the suspicion of exercising such power unfairly, and for sinister ends, will ever be entertained.

We must all know how possible it is, in the wide and almost boundless range of science which a perfect medical education comprises, to find some unfrequented track, which a candidate intent only on the plain straight forward road of his profession may have never travelled, and on which he may readily, by a wily examiner, be made to stumble. There seems no pretence, however, for such power being at all exercised by Colleges of Physicians, while many cogent reasons may be urged against it; and therefore there can be no doubt, in my mind, that it ought to be abolished.

In the internal regulation of these bodies, too, some amendments should take place. If the distinction of fellows and licentiates be necessary, and that it is deemed expedient to vest the corporate rights solely in the former, let them be prevented, as far as is practicable, from abusing the trust reposed in them; let them not have the power of reducing their numbers by neglecting to elect; nor of establishing a monopoly, by which private interests alone are promoted, while those of the public are trampled on and forgotten.

The preservative against this species of corruption is plain and obvious, and consists merely in rendering it obligatory on the fellows to keep up their number to a certain amount, and committing the election to vacancies entirely to the licentiates: these would be pretty sure not to fail in exercising this power when duly called on; or should they unaccountably accountably neglect their duty in this respect, it might then be competent to the fellows, after a given time had elapsed, to hold such election themselves. The body corporate can be considered in no light but as representing the body at large; and it is surely a solecism in representation for the representative body to be formed by means of election conducted by the representatives, and not by the represented.

It is quite unnecessary to pursue the subject, or to enter into more detail. From what has been already written, it must appear sufficiently plain what are the amendments necessary in the profession of physic, considering it as one comprehensive political institution, and also how they may be best effected. For some of them the interference of legislative power would doubtless be requisite, while many might be effected by mere arrangements on the part of our universities and medical corporations. Some there are which require only not to be impeded by improper interference, or inconsiderate legislation; and it was principally on account of these, which appeared to me to be in much danger from the well-meant but injudicious zeal of our reforming societies, and not with any vain hope of being able to organize anew so great and complex a body, that I was induced to bring forward the foregoing remarks: if they tend to introduce a juster way of thinking respecting this much-abused profession, and to convey juster and more distinct conceptions respecting the various characters that belong to it, I shall conceive myself repaid; my utmost expectations will be answered if they should have any effect in preventing those unwise measures about to be proposed to the legislature from receiving their sanction. I cannot think that the subject is at all prepared for undergoing a legislative discussion; and I am fully persuaded, that any such bill as the apothecaries are about to bring forward, must, if passed into a law, be productive of incalculable injury. My reasons for this opinion, and the principles which it is founded on, I have endeavored in the foregoing pages to place before the public as distinctly and forcibly as I can; and I now take my leave of the subject, in the hope that it will speedily attract the attention it deserves, and be discussed at greater length by some more able advocate.

One parting sentence more relative to the writer of these remarks, to whom views might otherwise be imputed which might tend to lessen his credit with the public, and possibly impair the force of his arguments; a mode of reasoning by no means unfrequent, and which it is deemed prudent thus to anticipate. He is not a surgeon, seeking to vindicate his encroachments on the province of physic; neither is he a surgeonapothecary, however earnestly he may have advocated the encouragement of this excellent species of practitioner. To physic alone does he belong, and he can appeal to the Editors of this Journal for the truth of these assertions. When the tenor of his remarks is recollected, he trusts he will stand acquitted of all undue bias towards his own particular department, and will be deemed justified by truth in subscribing himself *A disinterested Physician.*

---

*To the Editors of the Medical and Physical Journal.*

